# Intrinsically Disordered Regions May Lower the Hydration Free Energy in Proteins: A Case Study of Nudix Hydrolase in the Bacterium *Deinococcus radiodurans*

**DOI:** 10.1371/journal.pcbi.1000854

**Published:** 2010-07-15

**Authors:** Omar Awile, Anita Krisko, Ivo F. Sbalzarini, Bojan Zagrovic

**Affiliations:** 1 Mediterranean Institute for Life Sciences, Split, Croatia; 2 Institute of Theoretical Computer Science and Swiss Institute of Bioinformatics, Zürich, Switzerland; 3 Faculte de Medecine Paris Descartes, INSERM U1001, Paris, France; 4 Department of Physics, University of Split, Split, Croatia; 5 University of Vienna, Department of Structural & Computational Biology, Max F. Perutz Laboratories, Vienna, Austria; National Cancer Institute, United States of America and Tel Aviv University, Israel

## Abstract

The proteome of the radiation- and desiccation-resistant bacterium *D. radiodurans* features a group of proteins that contain significant intrinsically disordered regions that are not present in non-extremophile homologues. Interestingly, this group includes a number of housekeeping and repair proteins such as DNA polymerase III, nudix hydrolase and rotamase. Here, we focus on a member of the nudix hydrolase family from *D. radiodurans* possessing low-complexity N- and C-terminal tails, which exhibit sequence signatures of intrinsic disorder and have unknown function. The enzyme catalyzes the hydrolysis of oxidatively damaged and mutagenic nucleotides, and it is thought to play an important role in *D. radiodurans* during the recovery phase after exposure to ionizing radiation or desiccation. We use molecular dynamics simulations to study the dynamics of the protein, and study its hydration free energy using the GB/SA formalism. We show that the presence of disordered tails significantly decreases the hydration free energy of the whole protein. We hypothesize that the tails increase the chances of the protein to be located in the remaining water patches in the desiccated cell, where it is protected from the desiccation effects and can function normally. We extrapolate this to other intrinsically disordered regions in proteins, and propose a novel function for them: intrinsically disordered regions increase the “surface-properties” of the folded domains they are attached to, making them on the whole more hydrophilic and potentially influencing, in this way, their localization and cellular activity.

## Author Summary

Intrinsically disordered proteins and protein segments carry out a wide range of important biological functions despite their lack of permanent tertiary structure. Using advanced computational methods we study the biophysical properties of the intrinsically disordered regions in the enzyme nudix hydrolase from the desiccation- and radiation-resistant bacterium *D. radiodurans*. Interestingly, these regions are absent in homologue proteins in non-extremophile bacteria, suggesting that they might be involved in helping the key rescue-and-repair proteins in *D. radiodurans*, such as nudix hydrolase, adapt to the extreme absence of water. We show that the disordered regions in nudix hydrolase enlarge the overall surface of the enzyme, and most importantly, increase its overall affinity for water (i.e. its hydrophilicity). We suggest a novel hypothesis that this, indeed, may be the principal function of disordered regions in some cases: they increase the chances of the protein to be located in the remaining water patches in the desiccated cell, where it is protected from the desiccation effects and can function normally.

## Introduction

The dominant paradigm for describing the functioning of proteins is that of well-defined, structured molecular machines undergoing concerted, conformational changes while carrying out their function [1]–[3]. However, over the past few years it has become clear that the reality is much more complex, and that there are proteins that simply do not have a defined tertiary structure, and yet still carry out multitudes of different important functions. These intrinsically disordered proteins and protein segments (IDPs), also called “natively unfolded” or “natively disordered”, are by definition difficult to study using classical methods of structural biology, but they have in recent years received significant attention, largely due to two facts [4]–[14]. First, it has become clear that these proteins are extremely abundant. Using mostly bioinformatics approaches, it has been shown that in eukaryotes about 30% of all proteins are largely intrinsically disordered (ID) and that about 50% have long ID stretches [15]. Among signaling proteins, in particular, about 70% have long disordered segments [16]. These numbers are large: even if some of the predicted disordered regions actually prove to be structured, it is likely that IDPs in eukaryotes by far exceed, for example, the entire population of membrane proteins.

The second motivating factor is the fact that IDPs are involved in a host of extremely important cellular functions such as molecular recognition, assembly, protein modification and entropic chain functions [6]–[8], [11], [14], [17]. For example, in complex cellular activities such as cell signaling or regulation, it is often required that actions of many key molecular players be tightly controlled and coordinated through interaction and recognition based on unique identifying features [8], [18], which, in the case of signaling proteins, are often located in ID regions. When recognition of multiple binding partners and high-specificity/low-affinity binding is required, the choice on the molecular level often involves IDPs or ID regions [6], [7], [19]. Finally, large numbers of IDPs are known to be involved in human diseases such as cancer, neurodegenerative diseases, diabetes, cardiovascular diseases and amyloidoses [14].

Thus far, some of the biggest advances in the study of IDPs have been accomplished through bioinformatics and data mining approaches [11], [12], [20]–[24]. It has been shown that amino-acid sequences of IDPs tend to exhibit high hydrophilicity, low sequence entropy, and lack of the so-called order-promoting amino acids and bulky hydrophobic residues. Based on such features, several bioinformatics tools have been developed that can reliably predict the level of intrinsic disorder in a given sequence [11], [20]. Moreover, using mostly NMR, small-angle X-ray scattering, and different spectroscopic methods [5], [7], [25], basic structural features of IDPs have been elucidated, including their molecular size, level of structural heterogeneity, role of transient structure in coupled binding-and-folding events, aggregation tendencies and presence of persistent structure [6], [7], [25]. In particular, a combination of computer simulation and experimentally-determined restraints has provided some of the first ensemble-level pictures of IDPs [13], [25], [26]. However, compared with our knowledge of the structure and mechanism of ordered proteins, our understanding of IDPs is still extremely rudimentary and incomplete, primarily because of their structural and dynamic complexity. In the present study, we use molecular dynamics simulations and free energy calculations to focus on the role of IDPs in an extremophile bacterium: *Deinococcus radiodurans*.

*D. radiodurans* is a non-motile, non-spore-forming bacterium that belongs to the *Deinococcaceae* family [27], [28]. It is characterized by an extreme ability to withstand high doses of desiccation and ionizing radiation. For example, this bacterium can survive a dose of 5000 Gy of ionizing radiation, inducing more than 200 DNA double-strand breaks, with no effect on its viability [27], [28]. However, the molecular mechanisms underlying the high radiation resistance of *D. radiodurans* are thought to have evolved primarily as a side effect of mechanisms to counter extreme desiccation, as the bacterium thrives in dry, arid environments [27]. Over the years, evidence has accumulated suggesting that there exists no single, dominant mechanism responsible for the extremophilic nature of *D. radiodurans*, but that rather a combination of different mechanisms is at play [27], [28]. These range from passive structural contributions, such as the increased genome copy number [29], compact nucleotide organization [30], high intracellular concentration of the ROS-scavenger manganese [31], to active enzymatic repair mechanisms, including nucleotide and base excision repair and DNA double-strand break repair [27], [28], [32].

However important these mechanisms may be, it is also possible that the bacterium's proteome has undergone major structural adaptations in order to cope with environmental stresses. One potential strategy to address this possibility is to compare the proteome of *D. radiodurans* with those of its non-extremophile relatives and look for conspicuous differences. Recently, one of us has taken exactly this approach and focused on the presence and the putative biological role of ID regions in the proteome of *D. radiodurans* (Krisko et al., 2010, manuscript submitted to *Proteins: Structure*, *Function and Bioinformatics*). A subset of proteins in *D. radiodurans* was identified that contain highly hydrophilic stretches with low sequence complexity, indicative of intrinsic disorder, that are absent in non-extremophile homologues. Interestingly, this list includes a preponderance of housekeeping and rescue-and-repair proteins, including DNA polymerase III, a nudix hydrolase, rotamase, ABC transporters, adenine deaminase and LEA proteins. To further probe the significance of this finding, we here focus on a variant of nudix hydrolase, occuring naturally as a dimer, and analyze the properties of its ID regions. Nudix hydrolases [33] are a large class of enzymes present in all organisms that hydrolyze a wide range of pyrophosphates, including nucleotide di- and triphosphates, dinucleotides and nucleotide sugars. Importantly, some members of the family degrade oxidized nucleotides and in this way prevent potentially mutagenic effects these would have if incorporated into nucleic acids. Other family members regulate the concentration of metabolic intermediates and signaling molecules [33]. Interestingly, *D. radiodurans* exhibits 26 different types of nudix hyrolase, which is about three times more than what would be expected given the size of its genome, and is the highest number per Mbp of any bacterial genome known. In particular, it has been reported that the majority of *D. radiodurans* nudix genes are strongly induced during the stationary phase, which has been implicated in metabolic reprogramming.

IDPs, such as the tail regions of nudix hydrolase, are extremely dynamic and are very difficult to study by X-ray crystallography or nuclear magnetic resonance (NMR). However, atomistic computer simulations and molecular dynamics (MD) techniques are ideally suited to provide a complete, atomistic picture of the diverse, dynamic ensembles characterizing the IDPs. Here, using sequence analysis methods and molecular dynamics simulations in conjunction with hydration free energy calculations, we show that the ID regions in this protein significantly alter its solvation properties, making the whole protein significantly more hydrophilic. We hypothesize that this, in fact, is the principal functional role of these ID regions, and that they have evolved to keep the key housekeeping and repair enzymes solvated, and consequently functional, under extreme desiccation conditions.

## Results

**Figure 1:**
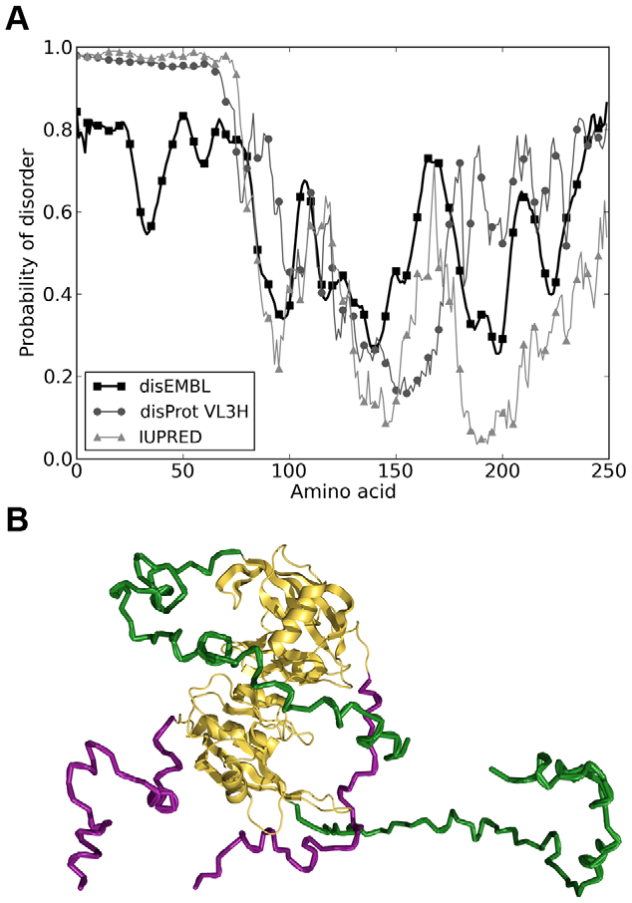
Prediction of disordered regions and a representative structure of DRNH. A) Disorder prediction using IUPRED, DisProt, and disEMBL algorithms. B) A representative simulated structure of a nudix hydrolase dimer with the N and C-terminal tails shown in green and purple, respectively.

We have analyzed the propensity for intrinsic disorder of the UniProtKB Q9RWW5_DEIRA sequence of nudix hydrolase from *D. radiodurans* (denoted hereafter as DRNH), using several different algorithms for predicting intrinsic disorder in proteins (Figure 1). Despite their different heuristics for determining disorder, all predictors classify the N-terminal region of DRNH, comprising approximately the first 80 residues, as intrinsically disordered. In particular, both the IUPRED and DisProt predictors associate disorder probabilities of over 90% with all residues below 75, with DisEMBL following suit at a somewhat lower level of significance. This convergence of different intrinsic disorder prediction algorithms is not surprising, given the sequence properties of the N-terminal tail of DRNH. Namely, it features a preponderance of disorder-inducing amino acids, such as glycine and proline, and extremely hydrophilic, polar amino acids, such as arginine and lysine, all of which are strong determinants of intrinsic disorder.

Compared to the sequences of homologue nudix hydrolases in non-extremophile bacteria, the sequence of DRNH studied here possesses a 40-residue C-terminal tail in addition to the N-terminal tail. Unlike the N-terminus, the C-terminus exhibits weaker signatures of intrinsic disorder (Figure 1). While DisEMBL and DisProt assign appreciable levels of disorder to this region (>50%), the IUPRED predictor considers it to be significantly less disordered, except for the very last 10 amino acids or so. Secondary structure prediction of both tails suggests that they are either in disordered or in coil conformation. For example, prediction of α-helical content using AGADIR [34] suggests that neither region is appreciably helical (helical propensity less than 1% for both tails). Similarly, JPRED [35] secondary structure predictor assigns no major secondary structure content to the N-terminus, and only a short putative 8-residue helical stretch to the C-terminal tail (residues 221–228). Based on these predictions, we consider both tails to be largely disordered, with the caveat that the N-terminus is likely significantly more disordered.

There is no high-resolution structure of nudix hydrolase from *D. radiodurans*. However, there exists an X-ray structure of the core region of a highly homologous (E-value of 8e-05) nudix hydrolase from *T. thermophilus*, including all residues except the N-terminal 80 and C-terminal 40 residues of the *D. radiodurans* nudix hydrolase. Using homology modeling (see Methods), we have generated a model of the nudix hydrolase from *D. radiodurans* in its dimeric form, with the missing regions modeled in a physically realistic, yet extended conformation. This model was then used as a starting structure for simulating a set of 200 independent molecular dynamics (MD) trajectories, each initiated using a different random number seed, and each exploring the phase space independently. Due to their innate flexibility and heterogeneity, the properties of ID regions can only be described on the level of ensembles. Consequently, we have run 200 independent MD trajectories to sample the possible conformations of the nudix tails in low-viscosity implicit solvent to further enhance sampling.

**Figure 2:**
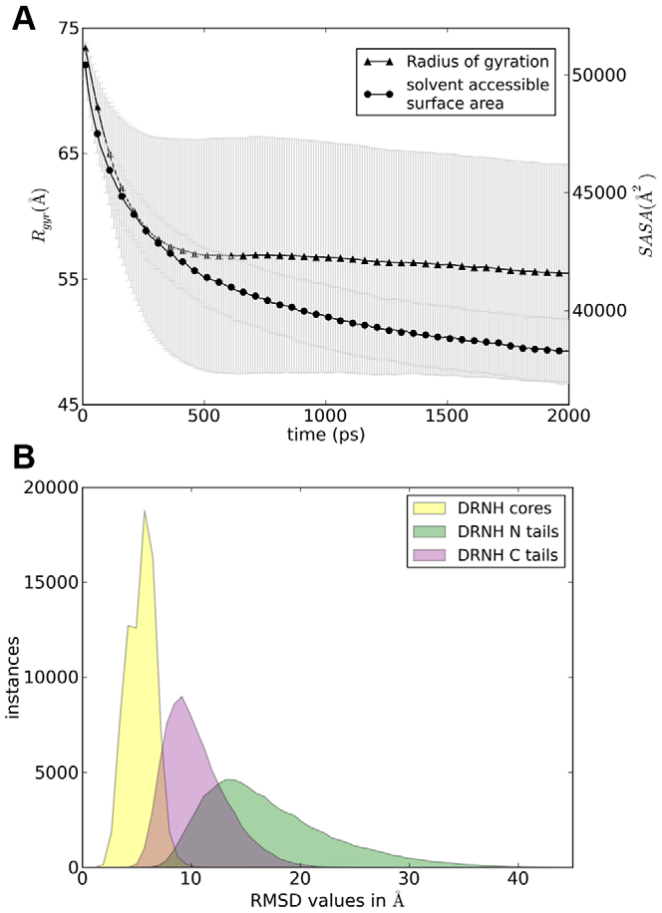
Structural characterization of DRNH trajectories. A) Radius of gyration and solvent-accessible surface area of nudix hydrolase trajectories as a function of time. B) All-against-all pair-wise RMSD distributions in Angstrom for the DRNH cores (yellow), N (green), and C-terminal (purple) tails.

As expected, the extended N- and C-terminal regions of the molecule collapse to a more compact form during the simulations, while the core domain remains largely unchanged. In Figure 2 we show the radius of gyration, *R_gyr_*, and the solvent-accessible surface area of DRNH as a function of time and averaged over all 200 independent trajectories. *R_gyr_* decreases monotonically, leveling out at about 550 ps, from which point on it decreases only slightly by another 2 Å. The standard deviation increases up to 10 Å during the simulation, indicating large variability in the generated trajectories. At 2 ns, the curves still exhibit a marginal downward trend, suggesting that some minor additional collapse may still be possible. Importantly, the collapsed structures of both tails are extremely varied, and share little structural similarity with each other. For example, the ensemble averaged pair-wise backbone root-mean-square deviation for the 200 final structures at 2 ns is 17.1±5.8 Å for the N-terminal 82 residues, and 10.4±2.8 Å for the C-terminal 40 residues, indicating significant structural diversity despite the compactness (Figure 2b). In contrast, the ensemble-average pair-wise backbone root-mean-square deviation for the dimer cores of the 200 final structures is 6.0±1.2 Å. However, if one focuses on individual cores and excludes the 5 relatively dynamic residues at either end of each core and a particularly mobile short loop (residues 147–151 in core 1 and residues 396–400 in core 2), these values drop to 3.7±1.0 Å (core1) and 4.4±0.8 Å (core2). This should be compared to the average backbone RMSD of 5.0±1.2 Å relative to the modeled starting structures of the cores, after the exclusion of the floppy termini and the mobile loop (see above). The latter value is relatively large compared to similar values of typical MD simulations, and can likely be explained by the expected structural relaxation away from the initially modeled structure. However, the secondary structure and the compactness of the core remain largely unchanged, suggesting that the resulting hydration free energies (see below) are not adversely affected by these changes. Together with *R_gyr_*, the solvent-accessible surface area of nudix hydrolase also decreases over time as the tails collapse and some of the initially exposed amino acids become buried (Figure 2).

As there is no experimental high-resolution information on the structure of DRNH, it is at present difficult to fully assess the accuracy of our simulated model of its unstructured tails, which is a general problem with most IDPs. To partially address this challenge, we have calculated the partial molar volume (PMV) of the simulated DRNH and its segments using the 3D-RISM formalism [36], [37], and compared it with calculated and experimental values for several different proteins (Figure S1). PMV is extremely sensitive to protein structure, and can be taken as a low-resolution test for the accuracy of our model [38]. Reassuringly, the PMV values for DRNH (full dimer, 41042±1825 cm^3^/mol) and its segments (N-tail single, 6579±43 cm^3^/mol; C-tail single, 3221±24 cm^3^/mol) fall closely on the line in the PMV versus molecular weight graph, defined by experimental as well as computed values for several different proteins (Figure S1). That many unstructured proteins have a similar partial specific volume as structured proteins (reflected in the linear dependence of the PMV on molecular weight) is a known experimental observation [39], and the fact that our results agree with it can be taken as partial evidence about the accuracy of our simulated model of DRNH.

**Figure 3:**
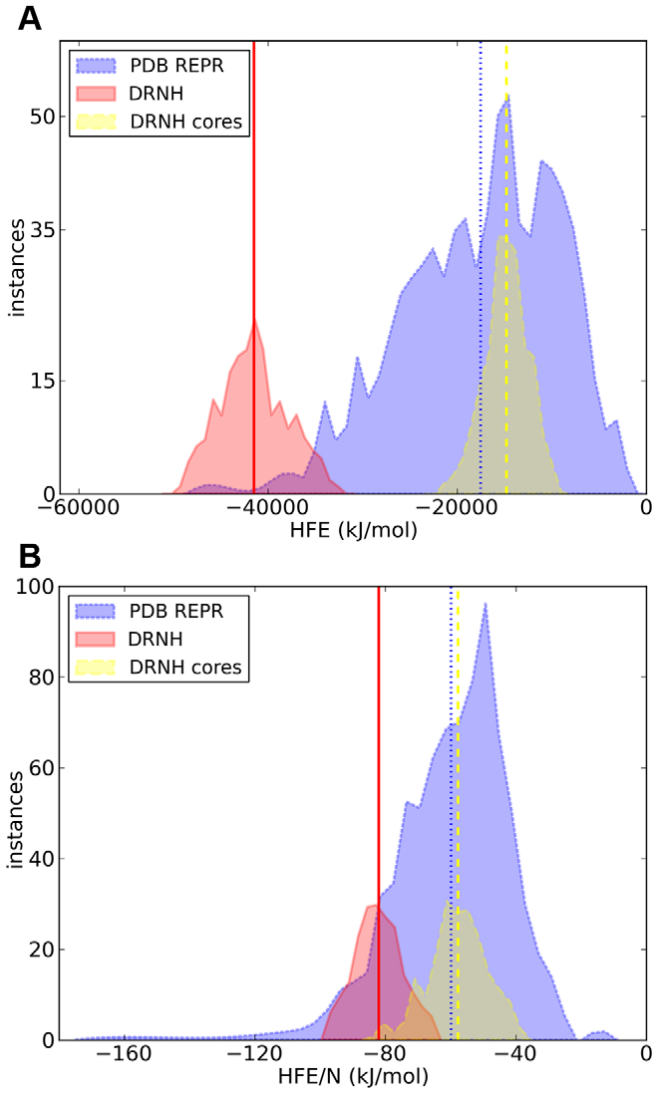
Analysis of hydration free energies of nudix hydrolase. A) Absolute and B) size-normalized hydration free energies of nudix hydrolase (red), nudix hydrolase without the N- and C-terminal tails (yellow), and the representative PDB set (blue). Distribution means (indicated by the vertical lines) and standard deviations in (a) are: for the representative set −17508±8219 kJ/mol, for DRNH −41009±3580 kJ/mol and for DRNH cores: −14794±2306 kJ/mol; in (b): for the representative set −59.77±18.58 kJ/mol/residue, for DRNH −82.02±7.16 kJ/mol/residue, and for DRNH cores: −57.79±9.01 kJ/mol/residue.

What is the influence of the nudix hydrolase tails on the solvation properties of the entire molecule? The set of structures resulting from our simulations was used to compare the absolute hydration free energy of nudix hydrolase with the hydration free energies of a set of representative reference structures from the PDB (Figure 3a). The hydration free energies (HFE) of proteins in the reference set are all below 0 kJ/mol and centered around moderate values (<HFE_ref.set_> = −17508±8219 kJ/mol) with a heavy tail in the direction of low hydration free energies. Since we excluded membrane proteins from the reference set, there are no structures with positive hydration free energies. Interestingly, the average hydration free energy of nudix hydrolase is more than 20000 kJ/mol below the average energy in the reference PDB set (−41009±3580 versus −17508±8219 kJ/mol). This is a significant deviation, with only very few proteins in the reference set being as hydrophilic as DRNH (Figure 3a). These results are reflected if one calculates the total free energy of the molecules, including their hydration free energy, but excluding their conformational entropy (Figure S2).

To test whether this large deviation in hydration free energy of nudix hydrolase is due to the ID tails, we created a set of structures with the N-terminal tail shortened by 44 residues from the N-terminus, and a set with neither N nor C-terminal tails at all. The hydration free energies of these structures show that a major contribution to the total hydration free energy of nudix hydrolase indeed comes from the two tails. For example, the average hydration free energy of the N-terminal Δ44 truncation mutants was −30438±3355 kJ/mol. Moreover, if one completely removes both ID tails of DRNH, its average hydration free energy is in the middle of the distribution of hydration free energies of the reference proteins (Figure 3a, “cores”).

The hydration free energy of proteins depends on their size. For this reason, we also compare the size-normalized hydration free energies of DRNH and the reference data set (Figure 3b). Albeit less dramatically, the size-normalized HFE of DRNH still remains significantly more negative than that of an average protein (with 90.5% of the reference proteins exhibiting a more positive hydration free energies than the average size-normalized HFE of DRNH).

**Figure 4:**
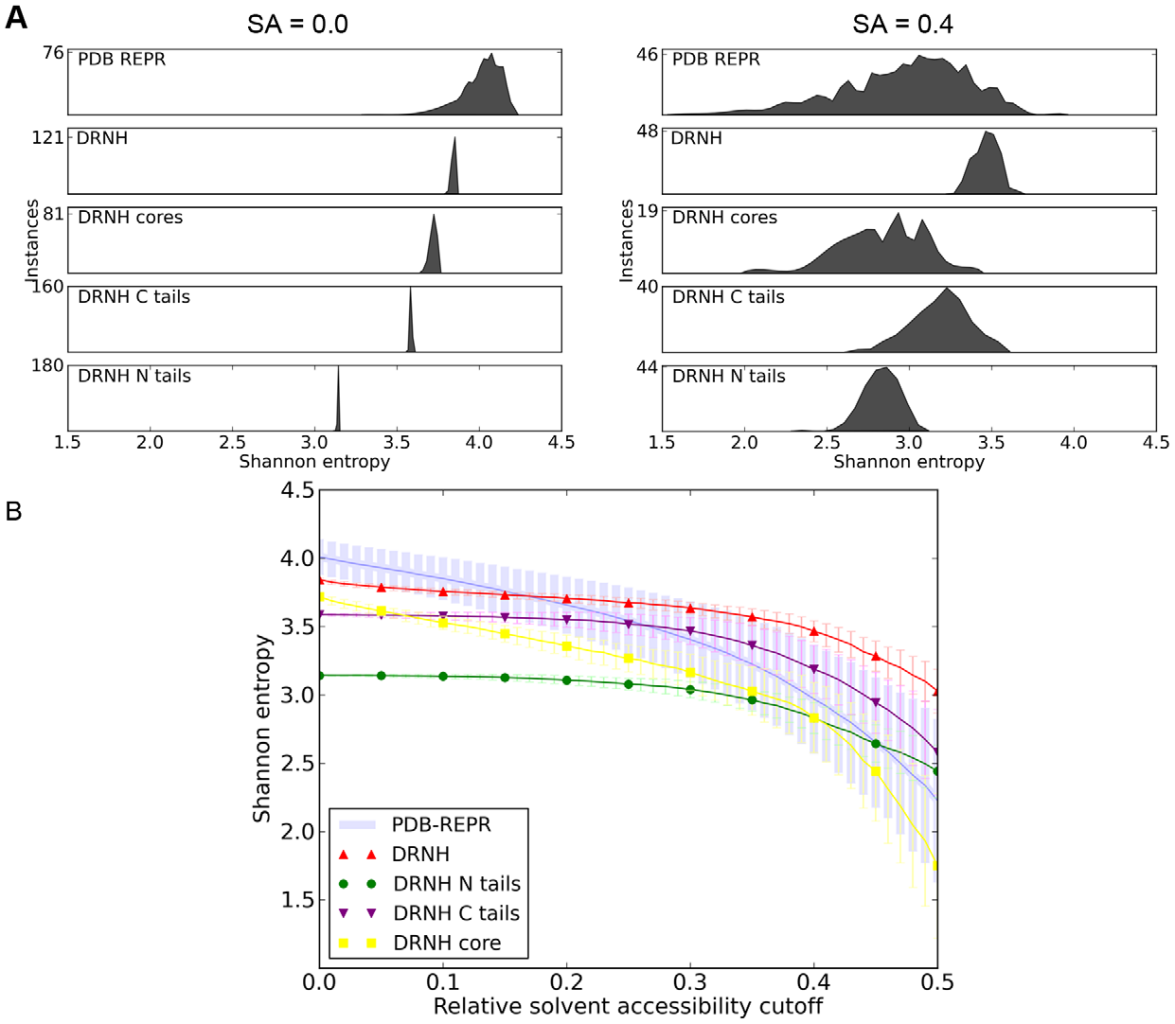
Analysis of sequence Shannon entropy of DRNH. A) Distributions of sequence Shannon entropy at solvent accessibility cutoffs of 0.0 and 0.4. B) Average sequence Shannon entropy at different solvent-accessibility cutoffs for nudix hydrolase, its core, tails, and the PDB representative set. The error bars are standard deviations from the average entropy of a given set.

Being extremely charged and hydrophilic, the composition of an ID stretch of amino acids is expected to share significant similarity with the composition of the water-exposed surface of a typical globular protein. In a way, one can think of ID regions as protein segments consisting of only surface and no hydrophobic core. How does this view agree with our simulated ensembles of nudix hydrolase from *D. radiodurans*? In Figure 4, we analyze the sequence complexity of different segments of DRNH as a function of relative solvent accessibility. In other words, we create mock sequences containing only those residues in the N-terminal tail, C-terminal tail, the core, or the complete DRNH sequence that are solvent-exposed to a given degree, and evaluate their sequence entropy (see Methods). Sequence entropy is a measure of the diversity (variability) of amino acids in a given sequence. In our case, it captures the heterogeneity of solvent-exposed residues in the tail and core regions of nudix hydrolase (the lower the value, the more homogenous are the types of residues on the surface). Importantly, the fact that all surface residues are strung together in one sequence plays no role – sequence entropy does not depend on the ordering of amino acids in a given stretch. We compare the complexity of nudix hydrolase' surface residue with the equivalent values obtained for the reference PDB set. First, looking at an accessibility cutoff of 0.0 (i.e. including all residues, both the buried ones and the ones at the surface), the sequence entropies of the complete N and C-terminal tails are significantly lower than that of either the complete nudix hydrolase core or the average protein from the reference PDB set. However, as one focuses more and more on just the exposed residues (i.e. with the accessibility cutoff increasing), the distribution of sequence complexities starts converging to a common value of about 3.0 at a solvent accessibility cutoff around 0.4 (Figure 4a). Importantly, this cutoff is the typical value accepted in the literature as a definition for surface residues. This means that if one looks at just the surface residues in the nudix core, or in a typical protein, their compositional complexity matches that of, for example, the complete nudix N-terminal tail. In other words, the complete N-terminal tail, by its sequence composition, is very similar to the surface of a typical globular protein. This trend is further exemplified in Figure 4b, where we show the dependence of the sequence complexities on the solvent accessibility cutoff.

## Discussion

The results presented in this paper show on several levels a correlation between the N and C-terminal tails' presence and an increased hydrophilicity of nudix hydrolase in *D. radiodurans*. We have shown a significant deviation of the tails' sequence complexity and hydrophilicity from those of the DRNH core. Furthermore, the significance of these results is enhanced by the fact that the tails' solvent-accessible area constitutes 64.91±1.41% of the total solvent-accessible area of the protein in the collapsed state. Finally, the results of the hydration free energy calculations show an even clearer picture: the hydration free energy of DRNH is twice as large as an average protein's hydration free energy, with the tails contributing the major part to this difference.

Although explicit-solvent molecular dynamics simulations and free energy calculations are typically more accurate, explicitly accounting for effects such as solute-solvent hydrogen bonds, the GB/SA implicit solvent [40]–[42] was the method of choice in our calculations for the following reason. Namely, implicit solvent methods are computationally less expensive and allow faster sampling of the conformational space, especially if performed at reduced values of solvent viscosity, as here. While the kinetic information coming from such simulations is likely compromised, the thermodynamic values, such as hydration free energies, should not be affected. Consequently, using implicit solvation allowed us to simulate a large system, which would hardly be accessible by any other means. In particular, ID regions are typically more flexible and less compact than average globular proteins, and therefore, at this time, implicit solvent simulations, along with Monte Carlo strategies, are likely the only feasible methods to study their structural and dynamic properties in atomistic detail. Finally, the 200-member set at 2 ns for a dimer of this size represents a state-of-the-art level of sampling on fast processors. Moreover, due to the solvent viscosity that is approximately 2 orders of magnitude lower than that of water, one can argue that the 2 ns trajectories actually capture events that would actually occur on the 200 ns time-scale. Namely, following the Kramers' relation, the rate of activated processes is inversely proportional to solvent viscosity in the high-friction limit [43]. While this relation does not hold for all values of solvent viscosity, it has been shown that for protein simulations it is valid for values down to approximately 1/1000 that of water [44], including the one used herein. That said, it should still be noted that implicit solvent models such as GB/SA may not be as accurate as explicit solvent models in capturing thermodynamic quantities such as hydration free energies [45]–[48]. In particular, it has been shown that the surface area dependent component in the GB/SA model, as well as in some other implicit solvent models, may not capture the non-polar part of the hydration free energy accurately enough for all applications in biomolecular simulations [49]–[51]. Most notably, the contributions of dispersion and solvent-accessible volume terms are missing in such models [52]. What is more, the accuracy of the GB/SA model critically depends on the accurate estimation of the effective Born radii [53]. An exciting novel approach for calculating solvation properties uses the three-dimensional reference interaction site model theory (3D-RISM) [36], [37], an integral equation theory for the behavior of molecular liquids, and has shown good agreement with other theoretical methods and experiments. Recent advances with this method even allow simulating molecular dynamics trajectories using the 3D-RISM formalism. It will be instructive to compare the results obtained here using the GB/SA formalism with those obtained using other methodologies. However, it is our belief that the quantitative effects observed in this study are so pronounced that they are independent of the potential inaccuracies in the specific methodology used for calculating hydration free energies.

While low viscosity has arguably allowed us to simulate events on the hundred nanosecond time-scale, it is still possible that with longer simulation time, one would observe major structural rearrangements of the tails. In particular, it is possible that some parts of the tails would actually adopt structured secondary and tertiary folds. This is particularly relevant for the C-terminal tail as its sequence exhibits lower levels of intrinsic disorder than the N-terminal tail. Would our conclusions be different if the tails adopted unique, ordered, folded structure? One way to address this question is to compare the hydration free energy of the complete nudix hydrolase dimer with those of similarly-sized folded globular proteins from our representative PDB set. In Figure S3, we summarize the results of such an analysis for nudix hydrolase and 23 globular proteins from our representative PDB set with sizes between 490 and 510 amino acids. Indeed, the hydration free energy of the nudix hydrolase dimer including the disordered tails is significantly more negative than those of the equivalent-size globular proteins (a difference of more than 5000 kJ/mol on average). In other words, if the tails were to adopt regular secondary and tertiary folds, it is likely that their hydration free energy would not be as negative as in the case for the unstructured tails. That said, any addition of extra hydrophilic amino acids, be it in the structured or unstructured conformation, would lower the hydration free energy of any protein. It is just that unstructured regions would likely have a stronger influence.

Note that the hydration free energy, as calculated and discussed here, is the free energy needed to transfer a protein from vacuum to water, and is only a first-order approximation to the actual free energy required to move a given protein from a desiccated region of a cell to a hydrated region. However, recalling the generalized Born equation, this approximation is likely only inaccurate up to a multiplicative constant. Namely, in the generalized Born formalism, the polar part of the hydration free energy of transfer between two media is proportional to the difference in the inverse dielectric constants of the two media. The part that depends on the environment is just a pre-factor dealing with dielectric permittivities. One can assume that these are the same for all proteins, provided they find themselves in similar environments both in water and in desiccated aggregates. In this sense, the relative ordering of HFEs that we calculated (water - vacuum) is the same as it would be in the case of the difference in HFE between water and desiccated aggregates. The only thing we assume here is that 
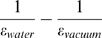
 has the same sign as 
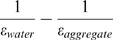
, which is likely the case, since aggregates are expected to have a lower permittivity than water. Another argument one can make is that in the GB/SA formulation, the environment enters the equations only through the dielectric permittivity, which one could claim to be similar in vacuum and in desiccated proteins. For this reason, we believe that the relative ordering of hydration free energies calculated here is the same as the ordering of free energies for transferring proteins from the aqueous environment into desiccated parts of the cell.

Our results show that the hydration free energy of DRNH is significantly more negative than that of a typical protein. This effect may be functionally important when water is scarce. Namely, under extreme desiccation conditions, *D. radiodurans* loses large amounts of water and experiences oxidative stress, causing a number of changes. The removal of the hydration shell from phospholipids in the membrane causes an increase in their phase transition temperature and their transition to gel phase at environmental temperatures [54]. In the cytoplasm, sugars such as trehalose can replace the shell of water molecules around proteins [55]. At environmental temperatures, this leads to the formation of a glassy matrix within cell (with mechanical properties of a plastic solid) with increased viscosity limiting in such way all processes that require diffusion and ensuring stability [56]. Proteins known to be expressed in stages of extreme desiccation are those belonging to the late embryiogenesis abundant (LEA) family of proteins [57]. Their function and mode of action are far from understood and call for further investigation. They are characterized with long stretches of highly hydrophilic and charged amino acid residues that are disordered in structure and are similar to the tails studied herein [57].

**Figure 5:**
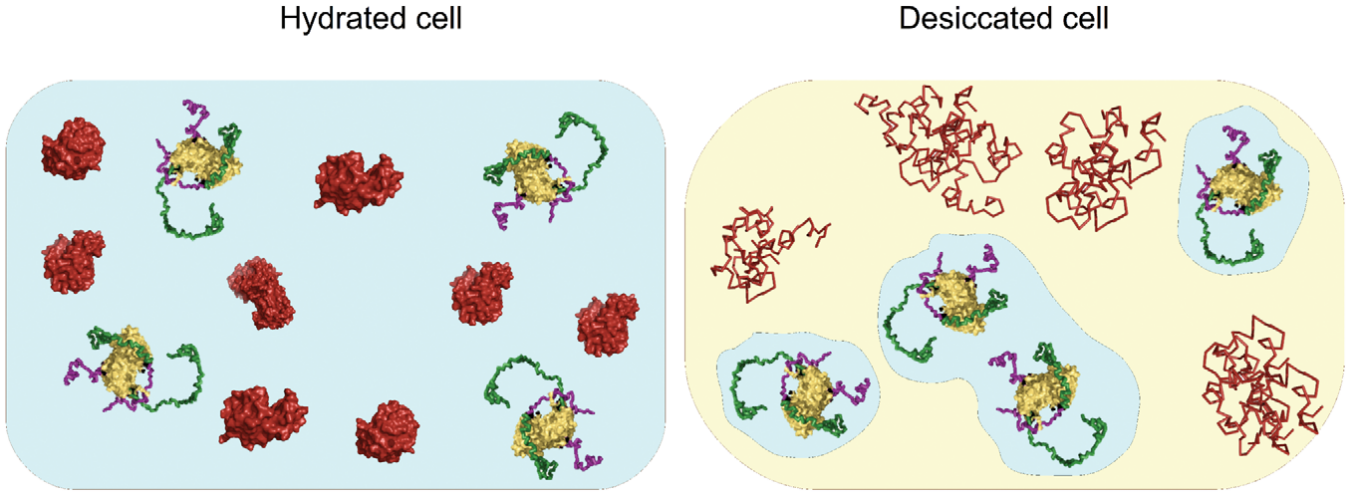
A hypothetical model of the behavior of nudix hydrolase in a hydrated and a desiccated D. radiodurans cell. (Left) A D. radioduranscell under physiological conditions, where all proteins are functional. (Right) The same cell in a state of desiccation: There are only patches of water left in the cell. Most proteins are not in aqueous milieu any more and are therefore denatured. Nudix hydrolase, however, having lower hydration free energy than most other proteins, is likely to be found in remaining patches of water and is hence still functional.

Under the conditions of extreme desiccation, it is important that housekeeping proteins and proteins required for efficient recovery after desiccation be additionally protected to help the organism recover in the first phase after rehydration. We hypothesize that precisely this is the function of the hydrophilic tails of *D. radiodurans'* nudix hydrolase – they increase the likelihood for the protein to stay in patches of residual water in the cell (i.e. in the more hydrated areas of the heterogeneous cellular matrix), while other proteins denature due to the removal of water (Figure 5). Note that this model, while supported by the data presented here, is preliminary and hypothetical – further experimental verification should shed light on its correctness. The ID regions at the termini of DRNH are unique to *D. radiodurans*, giving the bacterium a clear evolutionary advantage in harsh environments. One way to increase the overall hydrophilicity of a given protein is to change its surface residues in the course of an evolutionary process. However, if selective pressure on this trait is extremely strong, changing surface residues will at some point likely compromise the structural integrity of the whole protein. Therefore, we suggest that adding disordered tails at either the N or C-terminus may be a successful strategy to address this challenge without endangering the structural (and therefore enzymatic) integrity of the protein core.

IDPs have traditionally been associated with a number of particular functional classes: The function of entropic chains emerges completely from their lack of structure, effectors modify or inhibit the activity of their binding partners, scavengers store and neutralize small ligands, assemblers help assemble and stabilize protein complexes, and display sites mediate regulatory posttranslational modifications, such as phosphorylation or limited proteolysis [4]. Following the findings of the present work, we propose a new functional class for IDP regions, namely that of *hydrators*. The ID regions increase the probability for a protein to remain solvated in case of dehydration. Finally, as this function can be understood as a general mechanism for protecting proteins from denaturation, it can also be classified as a generalized chaperone activity. Future experimental and theoretical research should demonstrate the functional biological relevance of this hypothetical proposal.

## Methods

### Sequence-based analysis and 3D structure modeling of nudix hydrolase

There is no high-resolution structure of the nudix hydrolase studied herein (UniProtKB accession number Q9RWW5_DEIRA). We looked for possible homologues by performing a BLAST [58] search using the non-redundant data set (nr). A known structure of nudix hydrolase in *T. thermophilus* (PDB code 1VC8) was thus identified as the closest homologue of nudix hydrolase in *D. radiodurans* and was subsequently used to model the structure of the latter using Modeller [59]. As input, Modeller was also provided with secondary structure assignments for 3 residues that do not exist in the 1VC8 sequence, but are located in the middle of segments with well-defined secondary structure, as follows: GLY 165: β-strand, ASP 171: β-strand, ARG 205: α-helix. The N-terminal and C-terminal tails, unique to DRNH, were initially assigned physically realistic extended conformations. The 3D structure generated in this way was further refined using SQWRL3 [60], to optimize the position of amino-acid side-chains, and used as a starting structure for molecular dynamics simulations.

ID regions in the DRNH sequence were determined using three different algorithms: IUPRED [61] in the long-disorder mode, DisEMBL [21] 1.5 using the loops/coils definition, and the neural-network-based DisProtVL3H [62].

### Molecular dynamics simulations and evaluation of hydration free energies

Nudix hydrolase was simulated in its naturally occurring form as a dimer using the Amber 9 molecular simulation package. After steepest descent energy minimization, we generated 200 independent trajectories, each 2 ns long, from the starting Modeller-generated model structure using implicit, low-viscosity solvent and the Onufriev et al. [41] generalized Born/surface area (GB/SA) model. The simulations were carried out using Langevin dynamics with a collision frequency of 1 ps^−1^, with each independent trajectory initiated from a different random number seed. A cutoff of 16.0 Å was chosen for all non-bonded interactions, and SHAKE bond-restraints were applied to all bonds. The hydration free energies in this study were calculated using the generalized Born/surface area method (GB/SA) in the Amber package with the same parameters as the simulations. All structural and thermodynamic analysis of nudix hydrolase was carried out on a composite set containing 200 final structures from 200 independent trajectories (at 2 ns).

### Evaluation of partial molar volumes

Partial molar volumes (PMV) of the set of 200 final structures of DRNH, as well as a set of 7 proteins with sizes ranging from 50 up to 602 amino acids, were calculated using the rism3d_pmv routine available in AmberTools 1.4. The routine was parameterized to use the Kovalenko-Hirate closure [63]. The 7 proteins were chosen from a larger reference PDB set (see below).

### Surface-based analysis

The absolute and relative solvent accessibilities (SA) of each amino acid in the DRNH sequence, as well as in the reference PDB set, were calculated using DSSP [64]. The relative SA was determined by dividing the absolute SA of a given residue by the SA of the completely hydrated individual amino acid. The computed values allowed us to determine surface-exposed and buried residues as a function of variable cutoff.

The local sequence complexity of surface residues was quantified by the Shannon entropy, defined as:
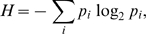
(1)where *p_i_* is the relative frequency of occurrence of each amino acid in a composite sequence containing all surface residues.

### Choice of the representative reference PDB set

In order to be able to compare the properties of nudix hydrolase in *D. radiodurans* to those of typical proteins, we selected and examined a set of representative proteins with known tertiary structure. Using the PDB-REPR [65] tool, we filtered the protein databank (PDB) (November 2007) for proteins whose structures were solved in X-ray experiments with a resolution ≤2.5 Å, an R-factor ≤0.3 Å, and having all side chains resolved. Furthermore, we did not allow structures with chain breaks, non-standard amino acids, and structures shorter than 40 amino acids. We also excluded all non-natural mutants of a given structure, complexed proteins, protein fragments, and membrane proteins. Finally, we manually verified the set and removed all misclassified structures and structures containing residues with more than one rotamer, thus obtaining a final set of 800 proteins, including multimeric ones. As a control, we compared the hydration free energies of this representative PDB set with the corresponding energies of the reference set of structures used by Feig et al. [66]. Tinker's [67] pdb2xyz and xyz2pdb utilities were used in preparing the Feig et al. [66] data set for Amber calculations.

Before evaluating any energies of the PDB reference set, we used Amber's molecular dynamics tool to minimize the potential energy of all structures, removing possible steric clashes due to different parameterizations used in the original PDB data.

## Supporting Information

Figure S1 Analysis of partial molar volume. Partial molar volume as a function of protein size, as calculated by the 3D-RISM formalism, of DRNH and its parts (squares), and compared with the calculated partial molar volumes of seven proteins from our representative PDB set (circles), and the experimentally measured partial molar volumes of five different proteins (crosses) (Imai et al. Chem. Phys. Lett., 395, 2004). The PDB codes of the proteins from the representative data set and their PMVs are: 1PTQ (4151.81 cm3/mol), 1UB9 (8458.46 cm3/mol), 1I39 (16735.06 cm3/mol), 1HCZ (20052.73 cm3/mol), 1RU4 (30743.60 cm3/mol), 1A8H (42650.21 cm3/mol), and 1EX1 (48276.45 cm3/mol). The proteins with experimental PMVs are BPTI (4690 cm3/mol), RNase A (9570 cm3/mol), Lysozyme (10080 cm3/mol), β-Lactoglobulin A (13690 cm3/mol) and α-Chymotrypsinogen A (18590 cm3/mol).
(0.09 MB TIF)

Figure S2 Analysis of free energy. Distributions of total free energy, including the GB/SA hydration free energy, but excluding conformational entropy, for the complete nudix hydrolase dimer (red, DRNH), the representative PDB set (blue, PDB REPR) and the core of the nudix hydrolase dimer (yellow, DRNH cores). Average values are shown as vertical lines.
(0.13 MB TIF)

Figure S3 A) Impact of disordered segments on the hydration free energy of proteins. A) Distributions of hydration free energy for the complete nudix hydrolase dimer (red, DRNH), a subset of structures in the representative PDB set with sizes between 490 and 510 amino acids (blue, PDB REPR) and the core of the nudix hydrolase dimer (yellow, DRNH cores). Average values of the distributions are shown as vertical lines. B) Ordered (DSSP categories helix and sheet) and disordered (DSSP categories turn and coil) secondary structure content for the complete nudix hydrolase dimer (red, DRNH), a subset of structures in the representative PDB set with size between 490 and 510 amino acids (blue, PDB REPR), and the core of the nudix hydrolase dimer (yellow, DRNH cores).
(0.28 MB TIF)
