# Supplementary figures and images for: Intrinsically Disordered Regions May Lower the Hydration Free Energy in Proteins: A Case Study of Nudix Hydrolase in the Bacterium Deinococcus radiodurans
Source: PLoS Comput Biol. 2010 Jul 15;6(7):e1000854. doi: 10.1371/journal.pcbi.1000854 (PMC2904767; doi:10.1371/journal.pcbi.1000854)

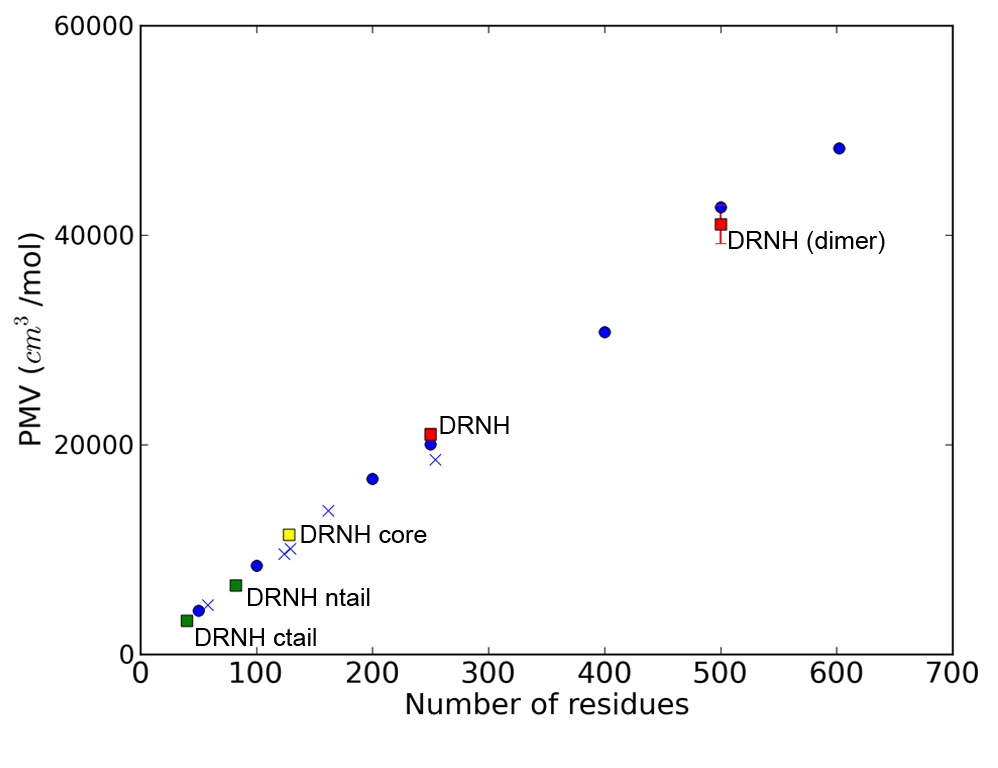

Supplement: Figure S1 — Analysis of partial molar volume. Partial molar volume as a function of protein size, as calculated by the 3D-RISM formalism, of DRNH and its parts (squares), and compared with the calculated partial molar volumes of seven proteins from our representative PDB set (circles), and the experimentally measured partial molar volumes of five different proteins (crosses) (Imai et al. Chem. Phys. Lett., 395, 2004). The PDB codes of the proteins from the representative data set and their PMVs are: 1PTQ (4151.81 cm3/mol), 1UB9 (8458.46 cm3/mol), 1I39 (16735.06 cm3/mol), 1HCZ (20052.73 cm3/mol), 1RU4 (30743.60 cm3/mol), 1A8H (42650.21 cm3/mol), and 1EX1 (48276.45 cm3/mol). The proteins with experimental PMVs are BPTI (4690 cm3/mol), RNase A (9570 cm3/mol), Lysozyme (10080 cm3/mol), β-Lactoglobulin A (13690 cm3/mol) and α-Chymotrypsinogen A (18590 cm3/mol). (0.09 MB TIF) [file pcbi.1000854.s001.tif]

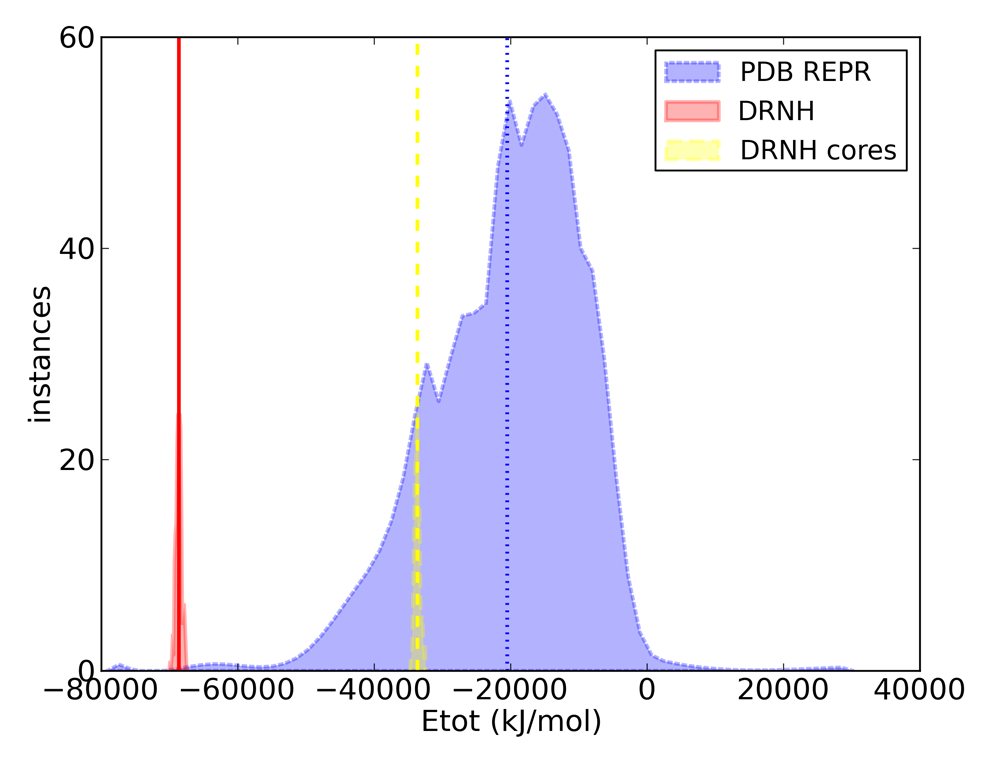

Supplement: Figure S2 — Analysis of free energy. Distributions of total free energy, including the GB/SA hydration free energy, but excluding conformational entropy, for the complete nudix hydrolase dimer (red, DRNH), the representative PDB set (blue, PDB REPR) and the core of the nudix hydrolase dimer (yellow, DRNH cores). Average values are shown as vertical lines. (0.13 MB TIF) [file pcbi.1000854.s002.tif]

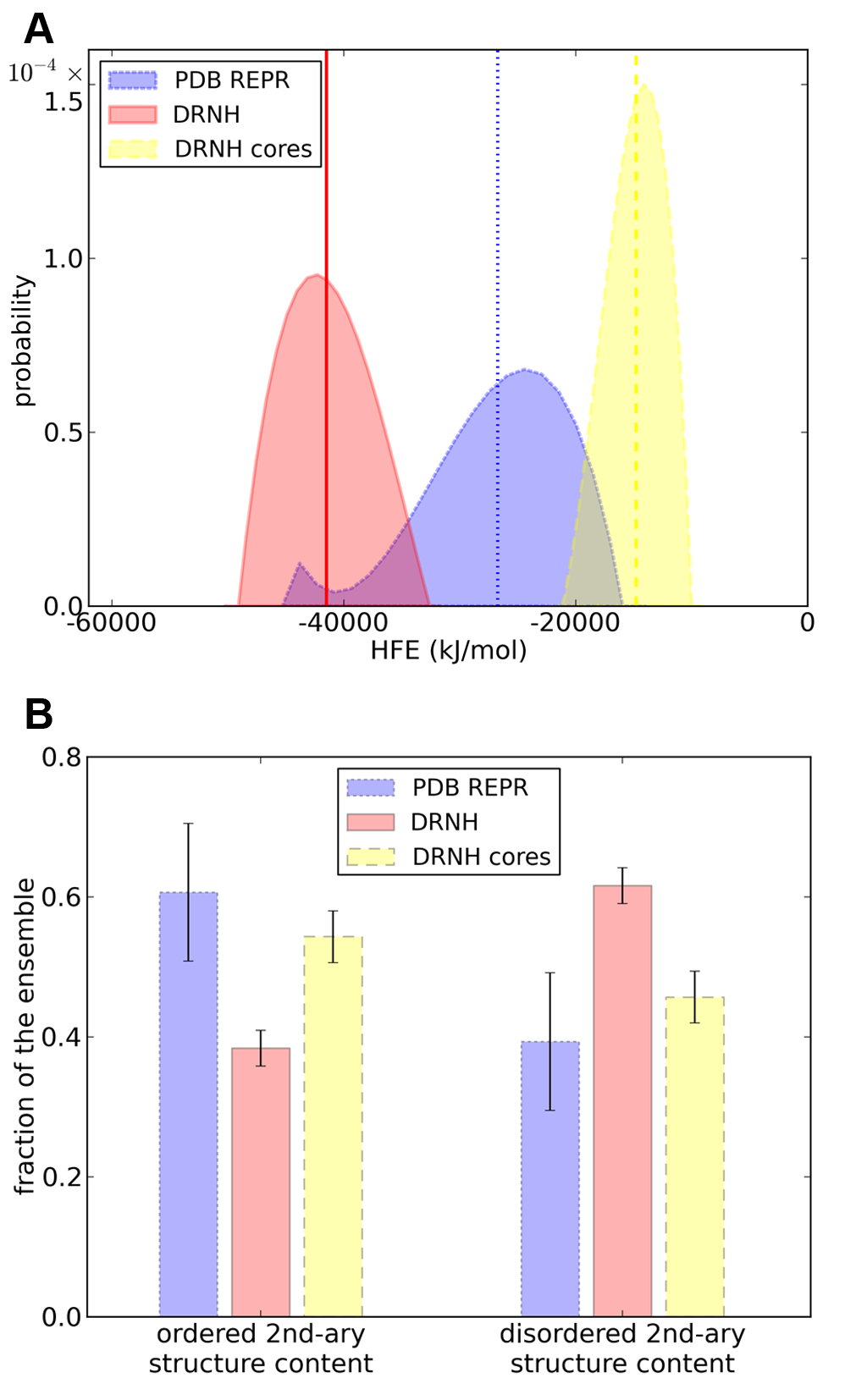

Supplement: Figure S3 — A) Impact of disordered segments on the hydration free energy of proteins. A) Distributions of hydration free energy for the complete nudix hydrolase dimer (red, DRNH), a subset of structures in the representative PDB set with sizes between 490 and 510 amino acids (blue, PDB REPR) and the core of the nudix hydrolase dimer (yellow, DRNH cores). Average values of the distributions are shown as vertical lines. B) Ordered (DSSP categories helix and sheet) and disordered (DSSP categories turn and coil) secondary structure content for the complete nudix hydrolase dimer (red, DRNH), a subset of structures in the representative PDB set with size between 490 and 510 amino acids (blue, PDB REPR), and the core of the nudix hydrolase dimer (yellow, DRNH cores). (0.28 MB TIF) [file pcbi.1000854.s003.tif]
